# Assessment of knowledge and attitude trends towards antimicrobial resistance (AMR) among the community members, pharmacists/pharmacy owners and physicians in district Sialkot, Pakistan

**DOI:** 10.1186/s13756-019-0517-3

**Published:** 2019-04-24

**Authors:** Hassan Waseem, Jafar Ali, Fiza Sarwar, Aroosa Khan, Hamza Saleem Ur Rehman, Mishal Choudri, Nooh Arif, Muhammad Subhan, Aansa Rukya Saleem, Asif Jamal, Muhammad Ishtiaq Ali

**Affiliations:** 1 Department of Biotechnology, University of Sialkot, Sialkot, Punjab 51310 Pakistan; 2 Department of Environmental Engineering, Michigan State University, East Lansing, Michigan 48823 United States; 3 Environmental Microbiology Laboratory, Department of Microbiology, Quaid-i-Azam University, Islamabad, 45320 Pakistan; 4 Key Laboratory of Environmental Nanotechnology and Health Effects, Research Center for Eco-Environmental Sciences, Chinese Academy of Sciences, 18 Shuangqing Road, Beijing, 100085 China; 5 Department of Earth and Environmental Sciences, Bahria University, Islamabad, 44230 Pakistan; 6 Department of Statistics, University of Sialkot, Sialkot, Punjab 51310 Pakistan

**Keywords:** AMR, Antimicrobial stewardship, One health, Resistance

## Abstract

**Background:**

Antimicrobial resistance (AMR) is an emerging threat to public health worldwide. A significant evidence has suggested that the knowledge and attitude trends among the community, pharmacists and physicians can play a critical role in managing the ever increasing threat of AMR.

**Methods:**

A cross-sectional survey was performed using three specific self-administered questionnaires for community members, pharmacists/pharmacy owners and physicians on a randomly selected sample population of 473, 424 and 308 respectively. Bivariate and multivariate logistic regression and Pearson chi-square tests were performed during data analysis.

**Result:**

A response rate of 81.2% (*n* = 385), 37.7% (*n* = 160) and 53.9% (*n* = 166) was achieved for general community, pharmacists/pharmacy owners and physicians respectively. More than half of the community participants (55.6%; *n* = 214) possess poor knowledge of AMR. Furthermore, knowledge and attitude of the community participants were also found to be significantly correlated (r^2^ = 0.02) with each other. In 90.6% (*n* = 145) of the pharmacies included in the survey, a qualified pharmacist was not present at the time of the operations. Only 36.9% physicians (*n* = 60) knew about the environmental route of dissemination of AMR. Majority of the physicians agreed that AMR is a global problem and also recognize the need for initiating AMR stewardship programs.

**Conclusion:**

Our study will provide effective assessment and potential insights in designing tri-faceted interventions for rationalizing antibiotics consumption thus controlling the development and dissemination of AMR.

**Electronic supplementary material:**

The online version of this article (10.1186/s13756-019-0517-3) contains supplementary material, which is available to authorized users.

## Background

Antimicrobial resistance (AMR) is one of the most serious and urgent public health concerns which can negatively influence healthcare, veterinary, and agriculture worldwide [1]. The increase of AMR can endanger the therapeutic effectiveness of antibiotics, increase treatment failures and, as a result, lead to longer and more severe illness episodes with higher costs and mortality rates. AMR alone has been approximated to cause around ten million deaths worldwide [2]. World Health Organization (WHO) has already declared AMR a high priority issue to be resolved by collective global action [3].

Like the rest of the world, an upsurge in infections with resistant strains has been reported in Pakistan [4, 5]. The increased prevalence of AMR is likely to have a significant impact on health care and environmental systems. The inappropriate and excessive use of antibiotics are among the key factors for the increase and spread of resistance [6]. At present, Pakistan is the third highest consumer of antibiotics after India and China among low to middle-income countries. The rate of antibiotics consumption in Pakistan between 2000 and 2015 have increased by 65% from 0.8 to 1.3 billion defined daily doses (DDD) [7]. This continuous increase in antibiotic consumption is becoming a major healthcare challenge from AMR perspective. A WHO report has already ranked Pakistan among top 5 countries with the highest number of neonatal deaths caused by resistant bacteria [8].

Abuse of antibiotics may arise from a complex interaction between numerous factors, such as patients’ knowledge, beliefs, and attitudes towards antibiotic use, self-medication, patients’ expectations, and patients’ experience with antibiotics [9]. The role of pharmacists/drug stores in curbing the antimicrobial resistance is also highlighted in many reports [10–12]. Besides, other factors include prescribers’ knowledge and experience, diagnostic uncertainty, perceptions of patients about the patient-prescriber interaction, and insufficient patient education by physicians [13]. Situation analysis report on AMR in Pakistan have also identified irrational prescriptions, over the counter availability of antibiotics, polypharmacy, misleading advertisements and copious consumption of antibiotics as key challenges [14], making the issue a tri-faceted one, involving the consumer/patient, the pharmacist/pharmacy owner and the physician. Thus, the control of antibiotic utilization needs multifaceted interventions involving the knowledgeable public, motivated community pharmacists and engaged healthcare practitioners.

World Health Assembly had endorsed a global action plan on AMR in May 2015. Raising public awareness and improving understanding of AMR are the key strategic objectives of this comprehensive plan on AMR. Reports from the world health organization (WHO) have suggested the monitoring and educational interventions aimed at rationalizing the antibiotics prescription, disposal, and consumption to curb AMR [15]. In Pakistan, over the counter availability of most potent antibiotics and irresponsible use of antibiotics by patients makes the problem a tri-faceted one involving patients, pharmacists, and physicians. To the best of our knowledge, there has not been even a single study assessing the patients, physicians, and pharmacists’ knowledge and attitudes towards AMR in Pakistan. Thus the current study was designed to evaluate the knowledge, attitude, and behavior among the aforementioned three primary stakeholders in Sialkot, Punjab Pakistan.

## Materials and methods

### Sampling size and location

A quantitative and cross-sectional survey was performed in Sialkot, a district in northeastern Punjab with an area of 3016 km^2^ and an estimated population of 3,893,672 people [16]. The survey was conducted in the winters from November 2018 to January 2019. The study was conducted in accordance with the Declaration of Helsinki and national and institutional standards. An approval from the “Ethical Committee, University of Sialkot, Pakistan” was obtained before initiation of the study.

Sample size, for the population of 3,893,672, was calculated by using the Raosoft sample size calculator using a confidence interval of 95%. An expected response of 50% was selected as there was utterly no idea about the knowledge and attitude of general public based on the absence of similar studies about the perceptions, knowledge, and attitude of the community members from Sialkot and other cities of Pakistan. The sample size of 385 was selected for the community/consumers. A stratified sampling method was used to approach 476 community members to ensure 385 filled questionnaires. Community members belonging to educational institutes, housewives, businessmen and also from other professions working in the premises of district Sialkot were included in the study. A total of 424 registered pharmacies and 308 physicians currently practicing in Sialkot were also approached to participate in the study.

### Questionnaires development

A literature review of similar studies was conducted in order to identify potential questions for the questionnaires used in the study [11, 15, 17, 18]. Based on the literature search, community/consumer questionnaires were adapted in accordance with the local population. The final questionnaire consisted of 27 questions covering three major areas: i) social demographic characteristics; ii) knowledge about AMR and Hepatitis C disease and iii) attitude and history on antibiotic usage and disposal. The community questionnaire was also translated in Urdu (national language of Pakistan) for convenient and accurate data collection. The accuracy and meaning of the translated content were checked by ten community individuals having firm command on both languages.

Similarly, structured questionnaires were also designed for pharmacists/drug store owners and physicians. The questionnaires were first validated and tested for its’ readability by a group of physicians and pharmacists in a pilot study (10 physicians; 10 Pharmacists). Both the questionnaires were developed after an extensive literature review of the studies highlighting the role and attitude of pharmacists and healthcare professionals in the context of AMR [12, 17] and were adapted in the recommendations of the responses of the pilot study. Final questionnaires for pharmacists/pharmacy owners and physicians comprises of eleven specific questions each. Copies of all three questionnaires are available in the Additional file 1 (Additional File). A five-point Likert scale ranging from “strongly disagree” to “strongly agree” is used to measure the response of the study participants. The Participants who were included in the pilot study were excluded from the main study. Study participants were first informed about the purpose of the study, and those who gave written consent were given structured questionnaires. Participants had completed the questionnaires anonymously which were collected afterwards. Incentives of any kind were not offered during the distribution of the questionnaires. Completed questionnaires were manually checked to exclude the incompletely filled questionnaires.

### Reliability and scoring

The reliability of the data analyzed in our study was first assessed using Cronbach’s α test [19]. The test results of the three different data sets were found to be reliable i.e., 0.70, 0.73 and 0.71 for community, pharmacies, and physicians respectively. To make results more intelligible in the text and scoring, “strongly agree” or “agree” were classified as agreed and “strongly disagree” or “disagree” as disagreed. A scoring system was applied to gauge the level of knowledge and attitude of the study participants. The knowledge and attitude scores were calculated by adding the number of correct responses by the community participants. Participants were divided into two categories on the basis of the level of knowledge they possess. Those who have answered at least 4 correct answers out of 7 were categorized into high knowledge possessing group while all those having < 3 correct responses were placed in low knowledge possessing group. A total of 3 questions regarding the knowledge of “Hepatitis C” were also included in the survey for comparison purposes. Similarly, attitude score was categorized into three levels indicated by bad (0–4), average (5–7) and good (8–11).

### Data analysis

All the questionnaires were manually checked and incomplete questionnaires were not included in the final analysis. All the completed questionnaires were entered into Epi-data version 3.1 and the data was exported to SPSS version 20 for analysis. The results were summarized by mentioning percent distribution in different categories. Association of the sociodemographic characteristics of community respondents with the knowledge of AMR and attitude of antibiotic usage was evaluated by using the bivariate and multivariate logistic regression respectively. A bivariate logistic regression analysis was performed for knowledge because it has been categorized only into two levels (high and low), whereas a multivariate logistic regression model was used for attitude which has been divided into three different categories i.e., bad, average and good. Statistical significance of *p* < 0.05 was accepted in this study. In the bivariate logistic regression, the model was built for all the demographic variables i.e., gender, age, residence, occupation, and monthly income were included whereas in the multivariate logistic regression model only education and age were included. A simple linear regression analysis test between knowledge and attitude of the community members was also performed, and autocorrelation between the two variables was also evaluated by computing Durbin-Watson value. A Pearson Chi-Square test was also applied to test the correlation between the AMR knowledge and availability of the pharmacists.

## Results

A total of 385 community participants were included in the study. Of the 385 participants, 56.4% (*n* = 217) were females, 41.8% (*n* = 161) had college/ university level education and 33.5% (*n* = 129) were students (Table 1). A total of 55.6% (*n* = 214) of the community members under study were possessing low knowledge about AMR whereas 44.4% (*n* = 171) were categorized in high knowledge possessing group. The participants were also asked about another chronic and life-threatening disease Hepatitis C, and 67.0% (*n* = 258) people have answered both the relevant questions correctly. The distribution of community respondents according to the bad, average and good attitude was found to be 61.8% (*n* = 238), 35.1% (*n* = 135) and 3.1% (*n* = 12) respectively.

**Table 1:** Sociodemographic characteristics of respondent community members in Sialkot

Characteristics	Frequency	Percentage
Gender		
Male	168	43.6
Female	217	56.4
Age		
18–30	233	60.5
31–45	107	27.8
> 45	45	11.7
Residence		
Urban	265	68.8
Rural	120	31.2
Education		
No Formal Education	61	15.8
Primary	39	10.1
High School	124	32.2
Undergraduate (B.Sc.)	150	39
Postgraduate (M.Sc)	11	2.9
Occupation		
Businessman	31	8.1
Govt. Employee	21	5.5
Student	129	33.5
Housewife	80	20.8
Farmer	9	2.3
Daily Wage Laborer	4	1
Others	111	28.8
Monthly Income (PKR)		
< 30,000	119	30.9
30,000-90,000	180	46.7
> 90,000	86	22.3

About 20% (*n* = 80) of the community members in our survey have agreed on disposing of the antibiotics with household waste (Additional file 1: Table S1). Of all dependent variables, evaluated in bivariate logistic regression, only education (*p* < 0.05; exp.(B) = 1.275) was found to be significantly affecting knowledge score of respondents whereas in case of multivariate logistic analysis both age and education as dependent variables gave a perfect model fit (*p* = 0.169). The results of the full model shows an insignificant effect of the independent variables on the attitude score of the community respondents. However, when the correlation between knowledge and attitude of the respondents was evaluated by simple linear regression, a significant correlation (*p* = 0.002) was found between the two variables (r^2^ = 0.02). Moreover, Durbin Watson value (1.71) also confirmed that the data in the two variables was not auto-correlated.

A total of 424 pharmacies in the vicinity of Sialkot city were approached. Out of which only 160, with 37.7% of response rate, has participated in our study. Independently operated pharmacies included in the study were 93.8% (*n* = 150) whereas hospital associated pharmacies were only 6.6% (*n* = 10). Surprisingly in 90.6% (*n* = 145) of the pharmacies covered in the survey, a qualified pharmacist was not present at the time of operations. 86.2% (*n* = 125) of the non-qualified pharmacy owners have poor knowledge about AMR and its stewardship programs whereas 73.3% (*n* = 11) qualified pharmacist not only possess good knowledge of antibiotic resistance but also understands the necessity of the return unwanted medicine (RUM) and AMR stewardship. About three-fourths of the pharmacy owners 74.4% (*n* = 108) have agreed that patients demand antibiotics from them without the prescription and about 76.6% (*n* = 111) have also acknowledged the need for antimicrobial stewardship program in Sialkot (Additional file 1: Table S2). A correlation between the AMR knowledge and availability of the pharmacist was also evaluated by using the Pearson Chi-Square test. Null hypothesis (H_0_) was assumed i.e., no relationship exists between the availability of the pharmacist and AMR knowledge. The χ2 (0.05;1) value was found to be 30.850 (*p*-value = 0.000) which leads to rejection of null hypothesis i.e., both variables are dependent on each other and significant relationship is present between AMR related knowledge and availability of pharmacists.

A total of 308 physicians working in the premises of Sialkot city were approached in the current study. Out of 308, 53.9% (*n* = 166) have provided consent and agreed to participate in the study. A majority of physicians i.e., 83.1% (*n* = 138) recognize that AMR is a worldwide problem while a similar percentage of physicians i.e., 82.6% (*n* = 137) agreed that patients with common cold demand antibiotics from them. Although only 30% (*n* = 53) physicians had received any AMR related training in last three years but about three fourths (74.1%) of the total physicians felt the need for the educational/awareness programs for enhancing the knowledge about AMR in the community. When asked about the environmental spread of AMR, surprisingly, only 60 physicians (36.9%) knew about the environmental route of AMR dissemination (Additional file 1: Table S3).

## Discussion

This is a first comprehensive study to be conducted in region Sialkot and probably in Pakistan demonstrating the knowledge, attitudes, and role of three main players in the context of AMR. Our results would provide a snapshot of the knowledge, attitudes, and patterns of antibiotics consumption in Pakistan. This study will also aid in the strategic development of community educational campaigns under Pakistan’s national action plan on AMR [20]. It is also anticipated that our study will provide an adequate assessment and potential insights in designing tri-faceted interventions for promoting appropriate antibiotic usage, replenishing the knowledge gaps and correcting the attitudes as part of an effective drive against AMR.

Primary data about the antibiotic abuse and subsequent resistance is scarcely available in Pakistan. Lack of funding from government and other NGOs in this field is the most probable reason for this data scarcity. Excessive and unnecessary use of antibiotics in hospitals of Pakistan is already well known [21]. The government of Pakistan has formed an intra-sectoral committee on AMR in recognition of the severity of the problem [20]. The committee has recommended antimicrobial stewardship along with other necessary steps. But designing and implementing stewardship programs requires the assessment of the level of the knowledge which the general public already possesses. In our study, 55.6% (*n* = 214) of the total community participants had depicted low knowledge about AMR. This percentage was closed to the knowledge percentages reported in similar studies performed in Kuwait and Ethiopia [15, 18]. Community participants in our study were found to have less knowledge about the efficacy of antibiotics against flu, common cold, and viruses. Only about one-fourth of the total participants correctly disagreed with the statements that ‘Flu and Common Cold can be cured with a course of antibiotics’ (*n* = 94; 24.4%) and ‘Antibiotics are an effective therapy against viruses’ (*n* = 95; 24.7%). This shows confusion exists among the general community regarding the accurate knowledge about antibiotics efficacy which could have severe implications owing to over the counter access of antibiotics to general public due to the non-regulated drugstores and pharmacies. The significant difference in the knowledge about the AMR and hepatitis C in the general community member could be due to various hepatitis C related awareness campaigns in the region which have educated the general community about the risk, prevention, and treatment of the chronic disease [21, 22]. AMR centric stewardship interventions can similarly improve the knowledge and attitude of the community members about this global threat. Such AMR stweardship programs have already been successfully opted in developed countries like USA, China, Canada, and some European countries [23–25]. Additionally, the finding that patients knowledge of AMR tends to have an impact on the attitude of antibiotics consumption can be used in designing effective strategies to bring an appropriate change in the general public about the proper use of antibiotics.

Apart from the consumer, community pharmacists and pharmacy owners also has an important role in ensuring widespread dissemination of knowledge about the proper use and disposal of drugs. To our surprise, a qualified pharmacist was not present in majority of pharmacies at the time of data collection in Sialkot district. Pharmacy owners and non-qualified staff were found to be managing the prescriptions from physicians. This depicts the dearth of regulations in pharmacy stores particularly in Sialkot and generally in Pakistan. Absence of a licensed and qualified pharmacist at an operational pharmacy is also in negation of WHO guidelines [26]. The absence and shortage of pharmacists has also been reported previously for other developing countries including Malaysia, Ghana, India and Nepal [27–30]. Unregulated pharmacy stores in Pakistan can have great implications towards ineffectively dealing with health threats, like AMR, at the country level as identified by global health report of WHO [31]. National and provincial drug acts should be implemented in true spirit to stop the pharmacies operations without a qualified pharmacist.

Likewise, the role of physicians in controlling the antibiotics’ abuse and AMR spread is already established. In our study, majority of physicians were found to be well aware about the global threat of AMR and also recognizes that AMR guidelines designed specifically at local level would be more beneficial than the international ones. It was also highlighted in our study that physicians’ prescriptions are influenced by the patients demand of antibiotics. This behavior emphasize the need for auditing the antibiotic prescriptions in the health care facilities in general and investigating the consultation behavior. Instead of satisfying the patients by unnecessary prescriptions patients can always be appeased by making them understand their diseases [32].

Environmental dimension of AMR dissemination has recently gained much attention [33, 34]. Questions regarding the antibiotics disposal and/or return were included in all the three questionnaires to explore the knowldege regarding environmental route of AMR spread. One-fifth of the community members agreed on disposing off their antibiotics along with the household waste. Disposal of expired and unwanted medicine with general waste and sewerage have also been reported previously [35]. Antibiotics discarded in the household waste bins can end up in landfills and may contribute to the development of resistant bacteria [36]. Disposal of antibiotics and other drugs down in sinks and toilets can not only negatively influence the aquatic life but also increases the risk of ARGs selection and dissemination into the environment [37]. It is therefore extremely critical to ensure the proper and safe disposal of antibiotics. Three-fourth of the pharmacists and pharmacy owners recognized the need for the RUM programs. In many developed countries like USA, Australia, England etc., RUM programs exists for the safe return of antibiotics and other drugs [35, 38, 39]. A similar system should be introduced in Pakistan under the supervision of the government agencies which will direct the pharmacies to advertise and guide the return of unwanted medicines. Research on the reasons for medicine disposal would also be helpful not only in terms of gauging the rational prescribing practices of physicians but also in minimizing wastage of drugs. Lack of the knowledge about environmental or non-clinical route of AMR spread among a significant number of physicians has also highlighted the need of the educational seminars specifically emphasizing this important driving factor of AMR spread in a community.

Some limitations which our study possess must be taken into consideration when interpreting the findings and results for the development of broad-scale policy framework. The study was conducted in one of the most developed regions of province Punjab and, also the majority of the population included in the study had an urban background, so it doesn’t allow the generalizability of the results on the provincial and national scale due to demographic constraints. The under representation of the farmers and daily wage laborers and over representation of the students in the present study indicates selection bias. Although maximum efforts have been made to include representative samples but the self-administered questionnaire based study has its own limitations i.e., only persons having ability to read and write can be included in the study. This type of selection bias may affect the external validity in terms of generalization of the results to a diverse community. The cross-sectional nature of the study also highlights that the data is collected at one point in time and, therefore, the current study was not able to track any trends, patterns and/or fluctuations in the knowledge and attitude of respondents towards antibiotic consumption and AMR over time. Despite these limitations, the current findings will provide an update about the knowledge of antibiotic usage and awareness of AMR. Given the global recognition and threat of AMR, a similar study on a national scale in Pakistan among the public, physicians and pharmacists is recommended to provide a more broader and comprehensive picture of the current status of antibiotic usage and awareness status of AMR in Pakistan.

## Conclusions

Our study has highlighted the gaps in AMR related knowledge and attitude among the community members in district Sialkot, Pakistan. Less knowledge towards AMR in the general public seems to be a serious problem, negatively influencing the antibiotic consumption behavior. The dearth of governance in healthcare systems particularly in pharmacies needs to be addressed by formulating and implementing strict laws and policies ensuring the presence of pharmacists for regulating over the counter access of the potent antibiotics. The study acknowledges the challenge of dissemination of AMR by over the counter and non-regulated availability of antibiotics. The role of physicians is of tantamount importance in the community for controlling AMR spread, so healthy prescription practices should be followed by physicians. Regular monitoring and auditing of prescriptions can help in reducing the appropriate antibiotics consumption. The need for initiating antimicrobial stewardship & RUM programs at local or national levels, with the objective of curbing the dissemination of AMR, has also been realized.

## Additional file

Additional file 1: **Table S1**. AMR related knowledge and attitude of respondent community members in Sialkot. **Table S2.** AMR related knowledge and attitude of non-qualified pharmacy owners in Sialkot. **Table S3.** AMR related knowledge and prescription trends of physicians practicing in Sialkot. (DOCX 1591 kb)

## Abbreviations

AMR: Antimicrobial Resistance
ARGs: Antibiotic Resistance Genes
DDD: Defined Daily Doses
MICS: Multiple Indicator Cluster Survey
RUM: Return of Unwanted Medicines
WHO: World Health Organization
