# Supplementary material for: Assessment of knowledge and attitude trends towards antimicrobial resistance (AMR) among the community members, pharmacists/pharmacy owners and physicians in district Sialkot, Pakistan
Source: Antimicrob Resist Infect Control. 2019 Apr 24;8:67. doi: 10.1186/s13756-019-0517-3 (PMC6482541; doi:10.1186/s13756-019-0517-3)
Supplement: Supplementary file 1 — Table S1. AMR related knowledge and attitude of respondent community members in Sialkot. Table S2. AMR related knowledge and attitude of non-qualified pharmacy owners in Sialkot. Table S3. AMR related knowledge and prescription trends of physicians practicing in Sialkot. (DOCX 1591 kb) [file 13756_2019_517_MOESM1_ESM.docx]

**Table S1.** AMR related knowledge and attitude of respondent community members in Sialkot.

|  | Strongly disagree n (%) | | Disagree n (%) | Neutral n (%) | Agree n (%) | Strongly disagree n (%) |
| --- | --- | --- | --- | --- | --- | --- |
| Knowledge Based Questions | | | | | | |
| Correct Statements |  |  | |  |  |  |
| Antimicrobial resistance develops when antibiotics no longer work to treat an infection. | 15 (3.9) | 81 (21.0) | | 111 (28.8) | 134 (34.8) | 44 (11.4) |
| Antimicrobial resistance is a global problem. | 15 (3.9) | 71 (18.4) | | 114 (29.6) | 157 (40.8) | 28 (7.3) |
| Antimicrobial resistance/ Superbugs can be a cause of death. | 18 (4.7) | 62 (16.1) | | 199 (51.7) | 82 (21.3) | 24 (6.2) |
| Incorrect Statements |  |  | |  |  |  |
| Antimicrobial resistance is the tolerance level of the body after which our body gets used to the antibiotic. | 12 (3.1) | 81 (21.0) | | 110 (28.6) | 140 (36.4) | 42 (10.9) |
| Flu and Common Cold can be cured with a course of antibiotics. | 15 (3.9) | 79 (20.5) | | 118 (30.6) | 143 (37.1) | 30 (7.8) |
| MRSA is a type of cancer. | 12 (3.1) | 85 (22.1) | | 189 (49.1) | 79 (20.5) | 20 (5.2) |
| Antibiotics are an effective therapy against viruses. | 19 (4.9) | 76 (19.7) | | 156 (40.5) | 109 (28.3) | 25 (6.5) |
| Attitude Based Questions |  |  | |  |  |  |
| I have consumed antibiotics without a doctor’s prescription (ever)? | 18 (4.7) | 94 (24.4) | | 64 (16.6) | 179 (46.5) | 30 (7.8) |
| I feel I know when to use antibiotics (for at least diarrhea etc.). I don’t need prescriptions. | 15 (3.9) | 114 (29.6) | | 85 (22.1) | 141 (36.6) | 30 (7.8) |
| I have insisted for antibiotic prescription to the doctor (even once)? | 17 (4.4) | 124 (32.2) | | 61 (15.8) | 161 (41.8) | 22 (5.7) |
| If I feel better after a few days, I sometimes stop taking my antibiotics before completing the course of treatment. | 25 (6.5) | 129 (33.5) | | 60 (15.6) | 139 (36.1) | 32 (8.3) |
| I prefer to keep antibiotics at home in case there may be a need for them later | 28 (7.3) | 113 (29.4) | | 59 (15.3) | 151 (39.2) | 34 (8.8) |
| It is good to be able to get antibiotics from relatives or friends without having to see a medical doctor. | 41 (10.6) | 131 (34.0) | | 75 (19.5) | 117 (30.4) | 21 (5.5) |
| I prefer to be able to buy antibiotics from the pharmacy without a prescription. | 33 (8.6) | 133 (34.5) | | 69 (17.9) | 121 (31.4) | 29 (7.5) |
| I prefer to use an antibiotic if I have a cough for more than a week. | 31 (8.1) | 129 (33.5) | | 74 (19.2) | 134 (34.8) | 17 (4.4) |
| When I have a sore throat I prefer to use an antibiotic | 36 (9.4) | 128 (33.2) | | 80 (20.8) | 131 (34.0) | 10 (2.6) |
| I often dispose of my antibiotics along with the household waste. | 70 (18.2) | 164 (42.6) | | 71 (18.4) | 61 (15.8) | 19 (4.9) |
| I always complete the course of treatment with antibiotics even if I feel better. | 17 (4.4) | 121 (31.4) | | 55 (14.3) | 169 (43.9) | 23 (6.0) |

**Table S2.** AMR related knowledge and attitude of non-qualified pharmacy owners in Sialkot.

|  | Strongly disagree n (%) | | Disagree n (%) | Neutral n (%) | Agree n (%) | Strongly disagree n (%) | |
| --- | --- | --- | --- | --- | --- | --- | --- |
| Knowledge Based Questions |  |  | |  |  |  | |
| Correct Statements |  |  | |  |  |  |  |
| Antimicrobial resistance develops when antibiotic no longer work to treat infection. | 2 (1.4) | 36 (24.8) | | 0 (0) | 84 (57.9) | 23 (15.9) |  |
| Antimicrobial resistance is a global problem. | 1 (0.7) | 18 (12.4) | | 19 (13.1) | 90 (62.1) | 17 (11.7) |  |
| Antimicrobial resistance/ Super bugs can be a cause of death. | 4 (2.8) | 28 (19.3) | | 59 (40.7) | 34 (23.4) | 20 (13.8) |  |
| Incorrect Statements |  |  | |  |  |  |  |
| Antimicrobial resistance is the tolerance level of the body after which our body gets used to the antibiotic. | 10 (6.9) | 46 (31.7) | | 9 (6.2) | 72 (49.7) | 8 (5.5) |  |
| Flu and Common Cold can be cured with a course of antibiotics. | 18 (12.4) | 80 (55.2) | | 2 (1.4) | 40 (27.6) | 5 (3.4) |  |
| MRSA is a type of cancer. | 4 (2.8) | 13 (9.0) | | 109 (75.2) | 17 (11.7) | 2 (1.4) |  |
| Antibiotics are effective therapy against viruses. | 4 (2.8) | 57 (39.3) | | 8 (5.5) | 58 (40) | 18 (12.4) |  |
| Attitude Based Questions |  |  | |  |  |  |  |
| Patients demand antibiotics without prescription as well. | 16 (11) | 20 (13.8) | | 1 (0.7) | 93 (64.1) | 15 (10.3) |  |
| We tend to guide people about the proper usage of antibiotics if they demand a non-prescribed drug. | 2 (1.4) | 5 (3.4) | | 15 (10.3) | 85 (58.6) | 38 (26.2) |  |
| Any NGO/ Government sector have given training to us about AMR. | 38 (26.2) | 75 (51.7) | | 25 (17.2) | 6 (4.1) | 1 (0.7) |  |
| There is a need for the RUM and AMR stewardship programs in Sialkot. | 0 (0) | 6 (4.1) | | 28 (19.3) | 71 (49.0) | 40 (27.6) |  |

**Table S3.** AMR related knowledge and prescription trends of physicians practicing in Sialkot.

|  | Strongly disagree n (%) | | Disagree n (%) | Neutral n (%) | Agree n (%) | Strongly disagree n (%) |
| --- | --- | --- | --- | --- | --- | --- |
| Patients demand an antibiotic treatment for a common cold? | 7 (4.2 ) | 15 (9.0 ) | | 7 (4.2) | 71 (42.8) | 66 (39.8) |
| Do you think antibiotic-resistant infections could make medical procedures like surgery, organ transplants and cancer treatment much more dangerous? | 3 (1.8 ) | 8 (4.8 ) | | 5 (3.0 ) | 75 (45.2 ) | 75 (45.2 ) |
| Antimicrobial resistance is a problem worldwide. | 4 (2.4) | 8 (4.8) | | 16 (9.6) | 95 (57.2) | 43 (25.9) |
| Antimicrobial resistance is a problem in Sialkot | 2 (1.2) | 9 (5.4) | | 23 (13.9) | 86 (51.8) | 46 (27.7) |
| Antimicrobial resistance is a problem in my daily practice. | 16 (9.6) | 32 (19.3) | | 17 (10.2) | 80 (48.2) | 21 (12.7) |
| The development of a local guidelines would be more useful than the international ones for antimicrobial resistance. | 11 (6.6) | 31 (18.7) | | 23 (13.9) | 72 (43.4) | 29 (17.5) |
| Antibiotic guidelines and antibiotic committee (If present) are an obstacle more than a help to clinical care. | 13 (7.8) | 59 (35.5) | | 38 (22.9) | 44 (26.5) | 12 (7.2) |
| I feel the need for organization of educational/ awareness programs on antibiotic resistance in the community. | 6 (3.6) | 17 (10.2) | | 20 (12.0) | 74 (44.6) | 49 (29.5) |
| Patients’ demands for antibiotics contribute to overuse | 12 (7.2) | 35 (21.1) | | 23 (13.9) | 73 (44) | 23 (13.9) |
| During the last three year, I have received some teaching/ training on antibiotics resistance spread? | 37 (22.3) | 52 (31.3) | | 24 (14.5) | 43 (25.9) | 10 (6.0) |
| I know about the nonclinical/ environmental routes for the spread of ARGs | 16 (9.6) | 40 (24.1) | | 50 (30.1) | 47 (28.3) | 13 (7.8) |

**Questionnaire for Community Members**

**Socio-demographic Characteristics:** (Please check the appropriate response)

**Q1. Gender**

| Male | Female |
| --- | --- |

**Q2. Age**

| 18-30 | 31-45 | >45 |
| --- | --- | --- |

**Q3. Residence**

| Urban | Rural |
| --- | --- |

**Q4. Education**

| No formal education | Primary Education | High School | College/ University |
| --- | --- | --- | --- |

**Q5. Occupation**

| Businessman | Government Employ | Student |
| --- | --- | --- |
| House-wife | Farmer | Daily Wage Laborer |
| Other |  |  |

**Q6. Monthly income (PKR)**

| ≤30,000 | 30000-90000 | >95000 |
| --- | --- | --- |

**Knowledge based questions:**

**Q7. Antimicrobial resistance develops when antibiotics no longer work to treat infection**

| Strongly Disagree | Disagree | Don’t Know | Agree | Strongly Agree |
| --- | --- | --- | --- | --- |

**Q8. Antimicrobial resistance is the tolerance level of the body after which our body gets used to the antibiotic.**

| Strongly Disagree | Disagree | Don’t Know | Agree | Strongly Agree |
| --- | --- | --- | --- | --- |

**Q9. Antimicrobial resistance is a global problem.**

| Strongly Disagree | Disagree | Don’t Know | Agree | Strongly Agree |
| --- | --- | --- | --- | --- |

**Q10. Flu and Common Cold can be cured with a course of antibiotics.**

| Strongly Disagree | Disagree | Don’t Know | Agree | Strongly Agree |
| --- | --- | --- | --- | --- |

**Q11. MRSA is a type of cancer.**

| Strongly Disagree | Disagree | Don’t Know | Agree | Strongly Agree |
| --- | --- | --- | --- | --- |

**Q12. Antibiotics are effective therapy against viruses.**

| Strongly Disagree | Disagree | Don’t Know | Agree | Strongly Agree |
| --- | --- | --- | --- | --- |

**Q13. AMR/ Super bugs can be a cause of death.**

| Strongly Disagree | Disagree | Don’t Know | Agree | Strongly Agree |
| --- | --- | --- | --- | --- |

**Q14. Hepatitis C is the disease which can cause inflammation and infection of liver.**

| Strongly Disagree | Disagree | Don’t Know | Agree | Strongly Agree |
| --- | --- | --- | --- | --- |

**Q15. Hepatitis C can be a cause of death.**

| Strongly Disagree | Disagree | Don’t Know | Agree | Strongly Agree |
| --- | --- | --- | --- | --- |

**Q16. I know about Hepatitis C from the following source?**

| Doctors | Social Media | Print/Electronic Media | Friends | Awareness Campaign |
| --- | --- | --- | --- | --- |

**Attitude Based Questions:**

**Q17. I have consumed antibiotics without doctor’s prescription (ever once)?**

| Strongly Disagree | Disagree | Don’t Know | Agree | Strongly Agree |
| --- | --- | --- | --- | --- |

**Q18. I feel I know when to use antibiotics (for at least diahorrea etc.,). I don’t need prescription.**

| Strongly Disagree | Disagree | Don’t Know | Agree | Strongly Agree |
| --- | --- | --- | --- | --- |

**Q19. I have insisted for antibiotic prescription to the doctor (even once)?**

| Strongly Disagree | Disagree | Don’t Know | Agree | Strongly Agree |
| --- | --- | --- | --- | --- |

**Q20. I always complete the course of treatment with antibiotics even if I feel better.**

| Strongly Disagree | Disagree | Don’t Know | Agree | Strongly Agree |
| --- | --- | --- | --- | --- |

**Q21. If I feel better after a few days, I sometimes stop taking my antibiotics before completing the course of treatment.**

| Strongly Disagree | Disagree | Don’t Know | Agree | Strongly Agree |
| --- | --- | --- | --- | --- |

**Q22. I prefer to keep antibiotics at home in case there may be a need for them later**

| Strongly Disagree | Disagree | Don’t Know | Agree | Strongly Agree |
| --- | --- | --- | --- | --- |

**Q23. It is good to be able to get antibiotics from relatives or friends without having to see a medical doctor.**

| Strongly Disagree | Disagree | Don’t Know | Agree | Strongly Agree |
| --- | --- | --- | --- | --- |

**Q24. I prefer to be able to buy antibiotics from the pharmacy without a prescription.**

| Strongly Disagree | Disagree | Don’t Know | Agree | Strongly Agree |
| --- | --- | --- | --- | --- |

**Q25. I prefer to use an antibiotic if I have a cough for more than a week**

| Strongly Disagree | Disagree | Don’t Know | Agree | Strongly Agree |
| --- | --- | --- | --- | --- |

**Q26. When I have a sore throat I prefer to use an antibiotic**

| Strongly Disagree | Disagree | Don’t Know | Agree | Strongly Agree |
| --- | --- | --- | --- | --- |

**Q27. I often dispose of my antibiotics along with the household waste.**

| Strongly Disagree | Disagree | Don’t Know | Agree | Strongly Agree |
| --- | --- | --- | --- | --- |

**Questionnaire for Pharmacists/ Pharmacy Owners**

**Name Address of Medical Stores __________________________**

**Hospital/ Independent**

**Pharmacist Available**

**Yes/ No**

**Knowledge based questions: (**Please check the appropriate response)

**Q1. Antimicrobial resistance develops when antibiotics no longer work to treat infection**

| Strongly Disagree | Disagree | Don’t Know | Agree | Strongly Agree |
| --- | --- | --- | --- | --- |

**Q2. Antimicrobial resistance is the tolerance level of the body after which our body gets used to the antibiotic.**

| Strongly Disagree | Disagree | Don’t Know | Agree | Strongly Agree |
| --- | --- | --- | --- | --- |

**Q3. Antimicrobial resistance is a global problem.**

| Strongly Disagree | Disagree | Don’t Know | Agree | Strongly Agree |
| --- | --- | --- | --- | --- |

**Q4. Flu and Common Cold can be cured with a course of antibiotics.**

| Strongly Disagree | Disagree | Don’t Know | Agree | Strongly Agree |
| --- | --- | --- | --- | --- |

**Q5. MRSA is a type of cancer.**

| Strongly Disagree | Disagree | Don’t Know | Agree | Strongly Agree |
| --- | --- | --- | --- | --- |

**Q.6 Antibiotics are effective therapy against viruses.**

| Strongly Disagree | Disagree | Don’t Know | Agree | Strongly Agree |
| --- | --- | --- | --- | --- |

**Q7. AMR/ Super bugs can be a cause of death.**

| Strongly Disagree | Disagree | Don’t Know | Agree | Strongly Agree |
| --- | --- | --- | --- | --- |

**Attitude based questions:**

**Q8. Patients’ demand antibiotics without prescription as well.**

| Strongly Disagree | Disagree | Don’t Know | Agree | Strongly Agree |
| --- | --- | --- | --- | --- |

**Q9. We tend to guide people about the proper usage of antibiotics if they demand a potent antibiotic without prescription.**

| Strongly Disagree | Disagree | Don’t Know | Agree | Strongly Agree |
| --- | --- | --- | --- | --- |

**Q10. Any NGO/ Government sector have given training to us about AMR.**

| Strongly Disagree | Disagree | Don’t Know | Agree | Strongly Agree |
| --- | --- | --- | --- | --- |

**Q11. There is a need for the return unwanted medicine (RUM) and AMR stewardship program in Pakistan.**

| Strongly Disagree | Disagree | Don’t Know | Agree | Strongly Agree |
| --- | --- | --- | --- | --- |

**Questionnaire for Physicians**

(Check the appropriate response)

1. **Does patients demand an antibiotic treatment for a common cold?**

| Strongly Disagree | Disagree | Don’t Know | Agree | Strongly Agree |
| --- | --- | --- | --- | --- |

1. **Do you think antibiotic-resistant infections could make medical procedures like surgery, organ transplants and cancer treatment much more dangerous?**

| Strongly Disagree | Disagree | Don’t Know | Agree | Strongly Agree |
| --- | --- | --- | --- | --- |

1. **Antimicrobial resistance is a problem worldwide**

| Strongly Disagree | Disagree | Don’t Know | Agree | Strongly Agree |
| --- | --- | --- | --- | --- |

1. **Antimicrobial resistance is a problem in Sialkot?**

| Strongly Disagree | Disagree | Don’t Know | Agree | Strongly Agree |
| --- | --- | --- | --- | --- |

1. **Antimicrobial resistance is a problem in my daily practice.**

| Strongly Disagree | Disagree | Don’t Know | Agree | Strongly Agree |
| --- | --- | --- | --- | --- |

1. **The development of a local guidelines would be more useful than the international ones for antimicrobial resistance.**

| Strongly Disagree | Disagree | Don’t Know | Agree | Strongly Agree |
| --- | --- | --- | --- | --- |

1. **Antibiotic guidelines and antibiotic committee (If present) are an obstacle more than a help to clinical care.**

| Strongly Disagree | Disagree | Don’t Know | Agree | Strongly Agree |
| --- | --- | --- | --- | --- |

1. **I feel the need for organization of educational/ awareness programs on antibiotic resistance in the community.**

| Strongly Disagree | Disagree | Don’t Know | Agree | Strongly Agree |
| --- | --- | --- | --- | --- |

1. **Patients’ demands for antibiotics contribute to overuse.**

| Strongly Disagree | Disagree | Don’t Know | Agree | Strongly Agree |
| --- | --- | --- | --- | --- |

1. **During last 3 year, I have received some teaching/ training on antibiotics resistance spread?**

| Strongly Disagree | Disagree | Don’t Know | Agree | Strongly Agree |
| --- | --- | --- | --- | --- |

1. **I know about the nonclinical/ environmental routes for the spread of ARGs.**

| Strongly Disagree | Disagree | Don’t Know | Agree | Strongly Agree |
| --- | --- | --- | --- | --- |

**URDU QUESTIONNAIRE**

**
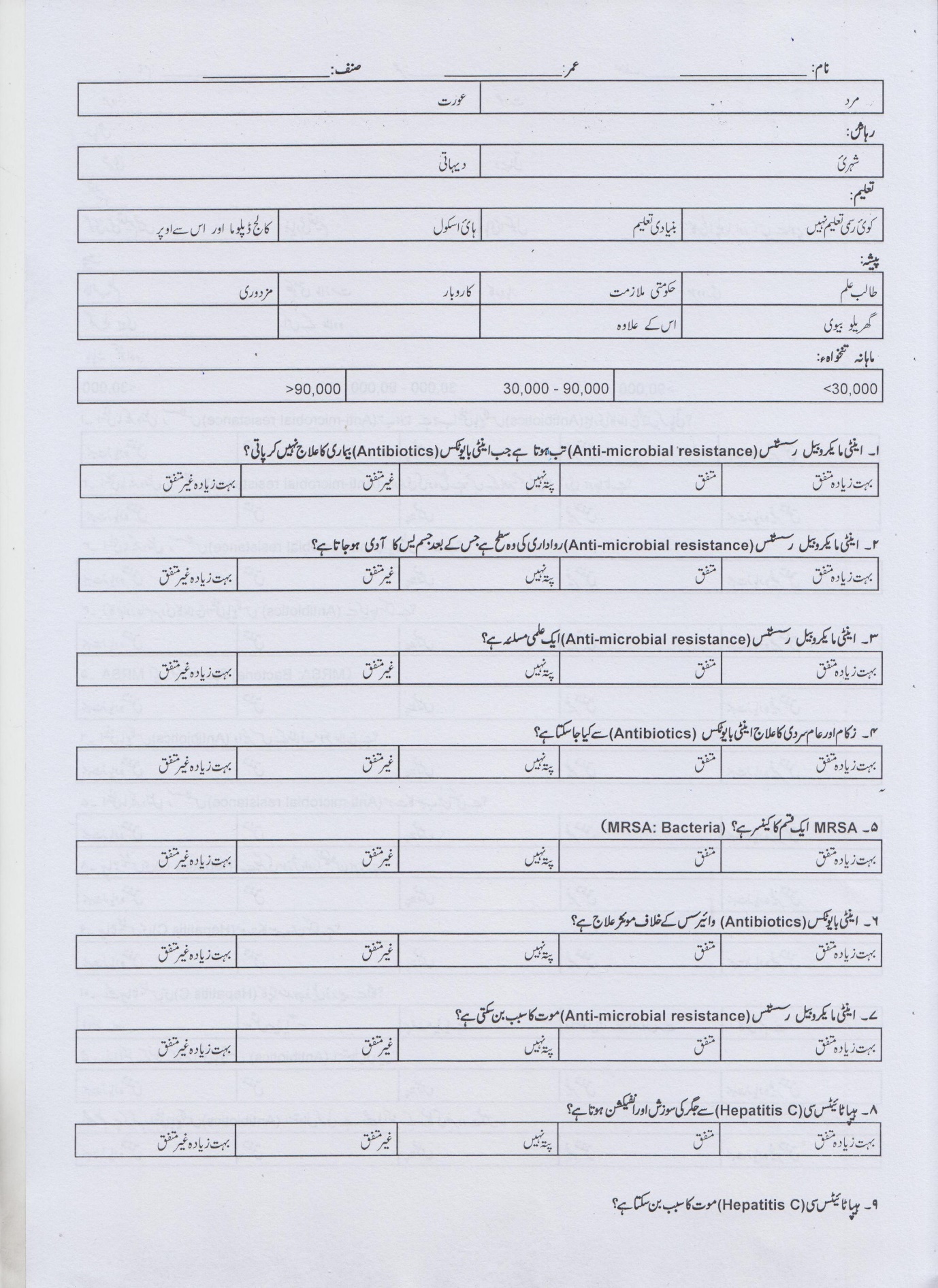
**

**
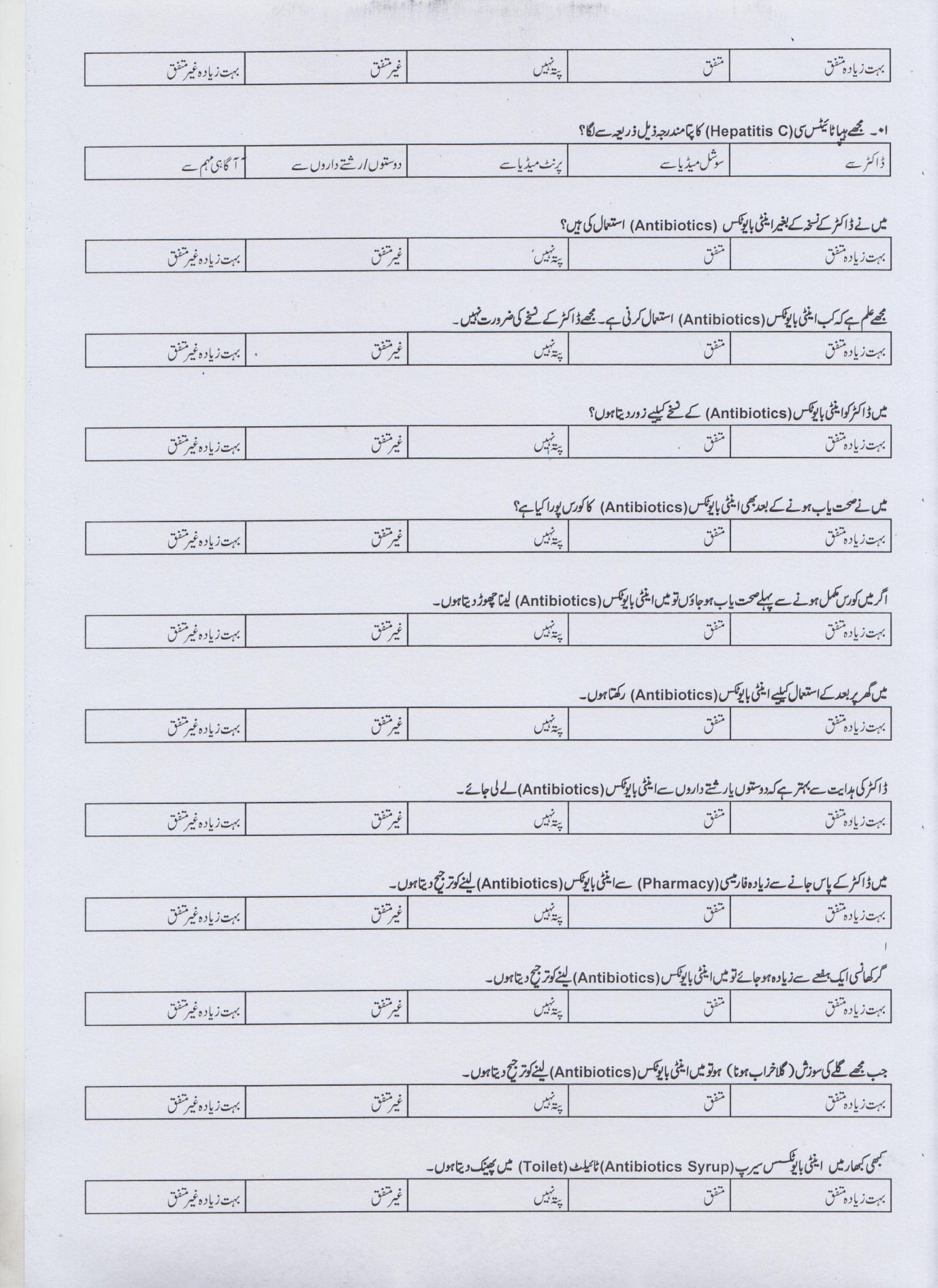
**
